# Hip Fragility Fractures: One Or Two Pathologies? A Systematic Review and Meta-Analysis of Demographic, Diagnostic and Therapeutic Aspects

**DOI:** 10.14336/AD.2025.0414

**Published:** 2026-07-25

**Authors:** José Luis Dinamarca-Montecinos, Miguel Yáñez, Ana Aguilera, Jean-Gabriel Minonzio

**Affiliations:** ^1^Faculty of Medicine, Universidad de Valparaíso, Viña del Mar, Chile.; ^2^Orthogeriatric Program, Adult Orthopaedics and Traumatology Ward, Hospital Dr. Gustavo Fricke, Viña del Mar, Chile.; ^3^Latin-American Academy of Medicine of the Older Adult (ALMA).; ^4^Interuniversity Network for Healthy Aging, Latin America and the Caribbean (RIES-LAC).; ^5^Doctorado en Ingeniería Informática Aplicada, Universidad de Valparaíso, Valparaíso, Chile.; ^6^Escuela de Ingeniería Informática, Universidad de Valparaíso, Valparaíso Chile.; ^7^Interdisciplinary Centre for Biomedical Research and Health Engineering “MEDING”, Universidad de Valparaíso, Valparaíso, Chile.

**Keywords:** Orthogeriatrics, osteoporosis, hip fragility fractures, intracapsular hip fractures, femoral neck fractures, extracapsular hip fractures, intertrochanteric fractures, subtrochanteric fractures

## Abstract

There is evidence that hip fragility fractures (HFF) are at least two different types of disease: intra- and extracapsular fractures (ICF and ECF). However, they are still mainly considered as one entity. Differentiating them may provide clues to improve their prevention, treatments and prognosis, and to reduce clinical, organisational and economic impacts. This work addressed published evidence about differences between ICF and ECF in older people, comparing demographic, etiologic, and therapeutic aspects, producing a summary of the state of the art, and determining which variables are associated with significant differences. A systematic review based on PRISMA methodology was conducted, searching in Google Scholar, Springer and Scopus from 01.01.1980 to 01.03.2024. Publications with *p-values* obtained from quantitative tests (*p* < 0.05 statistically significant) were included. For meta-analysis, Weighted Mean Method was used. 51 studies (19 countries, 5 continents, 129,075 subjects) were included. 78.4% of main objectives was searching for differences between both HFF. 60.8% provide evidence for demographic variables; 29.4% for diagnostic variables; 11,8% for therapeutic variables. ECF occurred at an older age (*p* < 0.05) in 43 studies (84.3%). There were no differences in sex (96.1%). 14 routine orthogeriatric blood parameters were studied. Haemoglobin, vitamin-B12, albumin and parathormone presented differences in >50% of the studies. Surgical management was significantly different in all studies. Significant demographic, diagnostic and therapeutic differences exist between ICF and ECF. There is a lack of studies combining variables, especially haematological exams.

## INTRODUCTION

Hip fragility fractures (HFF) have severe impact on functionality and quality of life, because of their association with significant comorbidity and mortality. Along with this, they impose a considerable financial burden, with direct and indirect costs rivalling the annual economic burden of cardiovascular disease [1]. A significant increase in its frequency and impact all over the world is projected in the coming decades [2]. Prevention of these fractures is therefore essential, and efforts to reduce their frequency have been primarily based on pharmacological secondary prevention strategies [3,4].

The main cause associated with HFF is considered to be osteoporosis, characterised by a decrease in bone mineral density. The drugs used to treat osteoporosis are effective in increasing bone mineral density. However, the goal of significantly reducing the frequency of HFF has not yet been achieved [3]. Attempts have been made to explain this failure by arguing that physicians rarely prescribe these medications for fear of their adverse effects (such as osteonecrosis of the jaw and atypical femoral fractures) [5], and that patients do not complete prescribed treatments due to discomfort in the administration of the drugs or due to adverse effects [3].

But there may be something else: For one nosological entity to be independent from another, there must be significant differences between them. These differences are generally found in anatomical, epidemiological, etiological, preventive, therapeutic, and evolutionary criteria [6]. And there is evidence that HFF could be at least two different types of disease.

Due to the presence of a synovial capsule—a specialised structure that encases the hip joint—hip fractures can be classified into intracapsular fractures (ICF) and extracapsular fractures (ECF): ICF correspond to fractures within the synovial capsule; and ECF correspond to fractures outside it. The intracapsular zone has a limited-but-enough bone arterial supply, while the extracapsular zone has a rich arterial supply [7].

Since the 60’s, there has been interest in elucidating whether ICF and ECF are distinct nosological entities, and several studies have been published showing differences in therapeutic (type of surgical treatment) and etiological aspects [6,8,9]. Etiology has been investigated using laboratory and imaging tests: Laboratory tests include routine, easily-access blood tests in hospital environment, related to the perioperative clinical workup [10-15]. There are also studies that include blood parameters linked to genetic research [12], but these are not easily accessible in the clinical setting. Imaging tests include radiological [16-18] and ultrasound [19] approaches. However, not all results are consistent, and some studies even have contradictory results. Furthermore, there are no reviews of the published work, so the differences found have not been analysed as a whole, and it has not been possible to reconcile the discrepancies that have arisen.

The difficulties and failures in prevention could be understood if ICF and ECF were two distinct nosologic entities. At the same time, this would support the search for differentiated preventive strategies, limiting objectives and costs. It would also provide greater support for differentiated clinical approaches, optimising outcomes for fractured patients, their families, healthcare teams, and healthcare institutions.

Although this is an important topic, there are no reviews that synthesise the evidence generated, and currently, HFFs are still understood, studied, and managed as if they were a single nosologic entity. Therefore, it is important to summarise the published literature, identify strengths and weaknesses, and lay the basis for further research.

The main objective of this review is to synthesise and compare scientific publication in this regard, using blood parameters, as these parameters are accessible, relatively inexpensive, and commonly requested in clinical settings. We will focus on related etiological variables, measurable by blood tests. We will refer to them as Chronic Risk Factors for Bone Damage (CRiF-BD). We will also consider some epidemiological and therapeutic variables.

## MATERIALS AND METHODS

### Ethics

This study was approved by the Institutional Bioethics Committee for Research in Human Subjects of the University of Valparaíso (CEC-UV) with resolution code: *CEC-UV 284-23*. The project and protocol of this Systematic Review and Metanalysis have been registered retrospectively in the Open Science Framework Platform under the following citation code: osf.io/nbvt8 and the following web direction: https://doi.org/10.17605/OSF.IO/2U67V

### Systematic Review Methodology

**Figure 1.**
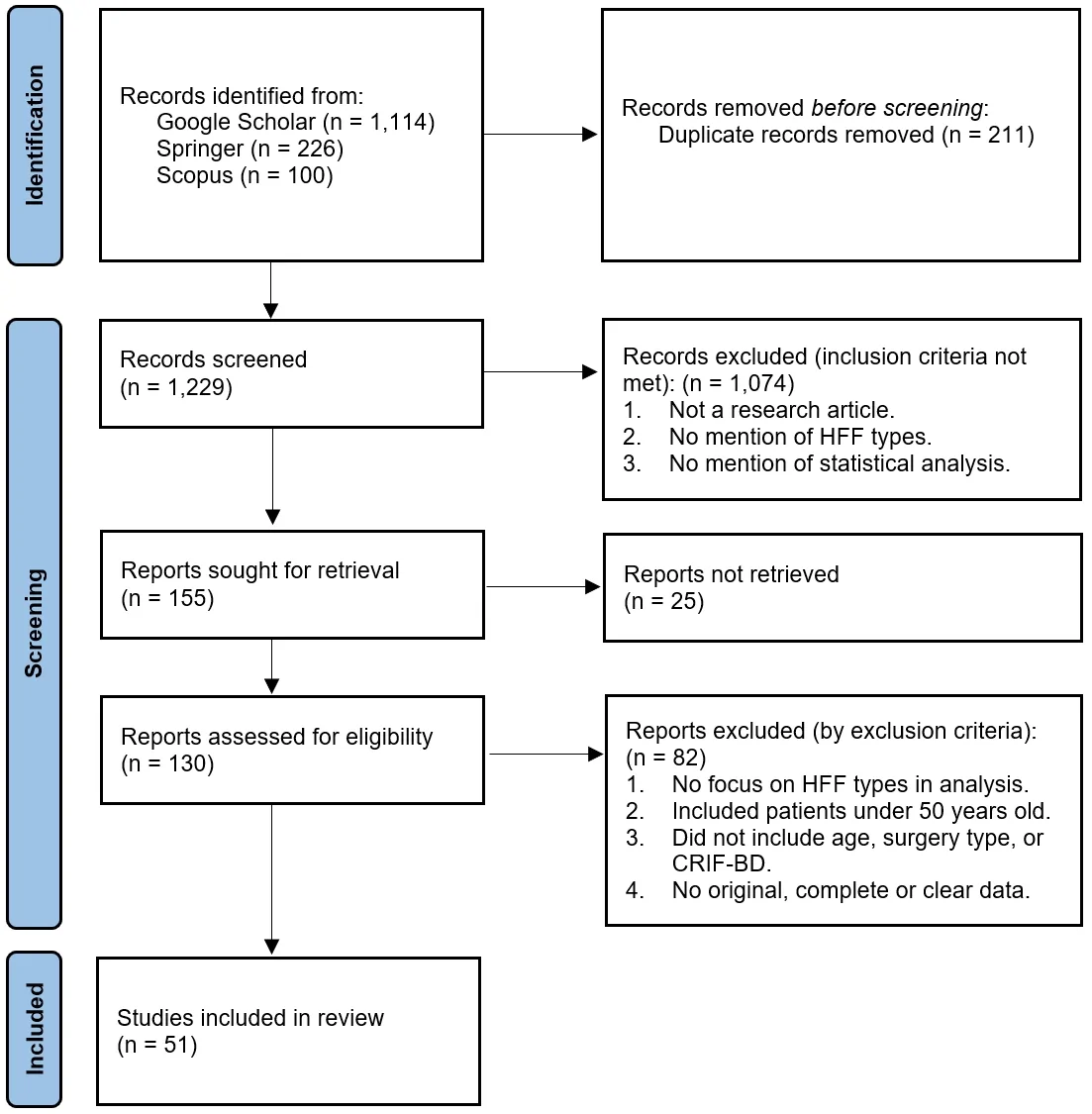
Flow diagram of the selection process.

The search and selection of recent and relevant studies to analyse was carried out following the PRISMA methodology (Preferred Reporting Items for Systematic reviews and Meta-Analyses) [20]. The general flow of the Systematic Review process is showed in Figure 1.

**Figure 2.**
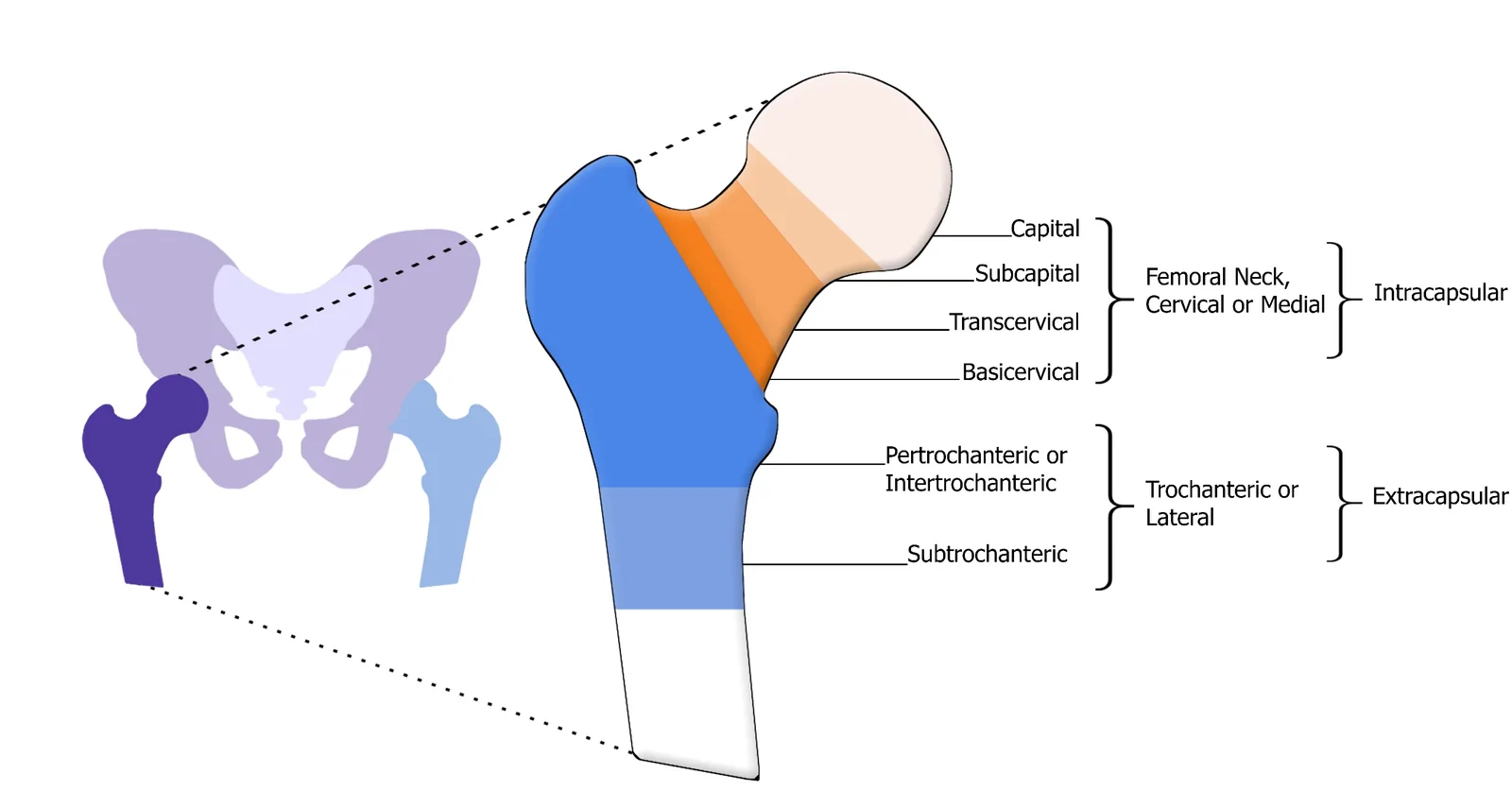
Anatomical classification of hip fragility fractures and correspondent synonymy.

Inclusion criteria: 1) Published research articles, such as cross-sectional, cohort, or case-control studies. 2) Their abstracts had to mention the types of HFF (intracapsular or extracapsular) or their synonyms (for ECF: intertrochanteric, pertrochanteric or lateral; for ICF: capital, subcapital, cervical, transcervical, or medial). For more clearness about this synonymy, Figure 2 should be consulted. 3) They also had to include details of at least one statistical analysis performed, the results of which were expressed as a p-value. 4) P-values had to be derived from quantitative tests such as the Mann-Whitney U test or the t-test, with p-values less than 0.05 being considered statistically significant.

The exclusion criteria were as follows: studies that did not focus their statistical analysis on ICF and ECF, studies involving younger patients (under 50 years old), and studies that did not include any of the following variables: age, surgery type, or CRiF-BD (uraemia, creatinine, INR, sodium, potassium, haematocrit, haemoglobin, platelets, vitamin D, vitamin B12, albumin, calcium, PTH, TSH, T4L, iron, transferrin, and transferrin saturation). Additionally, studies without original data or with incomplete or unclear data were excluded.

**Databases:** In March 2024, comprehensive searches were conducted in three databases: Google Scholar, Springer, and Scopus. The keywords used were ‘hip fracture type’, ‘intracapsular and extracapsular hip fractures’, and ‘trochanteric and cervical hip fractures’ (and the synonyms specified in Fig. 2). Due to the nature of the study, no date limit was set for the search.

**Selection process:** involved the abstracts reading, to determine their level of compliance with the inclusion criteria. This was performed by two researchers acting as independent reviewers. In case of difficulty in including or excluding an abstract, the final decision was made in agreement with the clinical researcher on the team.

**Data collection:** was performed manually. Reviewers collaboratively extracted and validated parameter values from patient reports, including their units of measurement, the statistical test used, and p-values. In addition, the number of patients, frequency of ICF or ECF, and terminology (synonymy) about type of hip fracture were considered. No automation tools were employed, except for data tabulations performed to facilitate synthesis and subgroup analysis, such as male-only or female-only cohorts. Regarding the terminology finally used to refer to HFF, we grouped the synonyms used in each study into intracapsular fractures (ICF) and extracapsular fractures (ECF), as appropriate (Fig. 2).

The variables studied were: 1) Bibliometric analysis, to measure the distribution of studies over time and across countries, and an analysis of whether the primary objective of each study was a comparison between ICF and ECF. 2) Demographic and epidemiological criteria, including geographic origin, sex, and age. 3) CRiF-BD for ICF and ECF, individually and in combination, according to their physiological relationships. 4) Type of surgical procedures performed for each type of HFF.

### Meta-analysis

The Weighted Mean Method was used, giving more weight to the studies that includes more subjects. The quantitative differences observed in the studies included from the review process were analysed to assess variations in specific values or measurements related to the subjects in those studies, referred to here as parameters. The analysis initially involved standardising all parameter values to a common measurement unit. This was followed by a visualisation of the generalised parameter values for HFF type by calculating a weighted mean of their values based on the number of included subjects in each study s∈S, denoted *Subjects_s_*, as follows:

$${\text{Weighted mean value}}^{p,f}=\frac{\sum_{s=1}^{S}{\mathrm{value}}_{s}^{p,f}\times {\mathrm{Subjects}}_{s}}{{\mathrm{Total}\;\mathrm{Subjects}}_{p}}$$ (1)

where *p* is the parameter index, *f* is the HFF type and *Total Subjects* is the total number of subjects dedicated to each parameter *p*.

After this, a trend for each parameter was identified based on whether each particular parameter was significantly lower, non-significant (NS), or higher in a given study with respect to the two HFF types. Subsequently, a majority trend was then drawn for a parameter based on the predominant opinion of all studies that included that specific parameter.

Finally, a percentage of agreement was assigned to each trend, and an overall agreement was defined for all parameters as follows:

$${\%\mathrm{Agreement}}_{p}=\left(\frac{{\mathrm{Mayority}\mathrm{Trend}}_{p}}{{\mathrm{Trends}}_{p}}\right)\times 100$$ (2)

$$\%\mathrm{Overall}\;\mathrm{Agreement}=\frac{\sum_{p=1}^{P}{\%\mathrm{Agreement}}_{p}\times {\mathrm{Trends}}_{p}}{\mathrm{Total}\mathrm{Trends}}$$ (3)

In Equation (2), the percentage of agreement for a parameter *p* is calculated as the proportion of majority trends (*Majority Trend_p_*) among all trends found for *p* (*Trends_p_*). Then, Equation (3) calculates the overall agreement by averaging the weighted agreement percentages for all parameters *P*. Each percentage is first multiplied by the number of trends for each parameter *p*. These weighted values are then summed up and divided by the total number of trends across all parameters (*Total Trends*) to reach the final overall agreement.

## RESULTS

### Review Process

This section starts with a description of the study selection process, followed by a disambiguation of HFF terminology. After it, the results of the systematic review are presented: the primary focus of the analysed studies, their bibliometric details, geographic distribution of fracture types, and the differences found in each parameter.

### Study Selection Process

Initially, 1440 studies were identified, from which 211 duplicates were removed, leaving 1229 for screening. The review of abstracts under inclusion criteria resulted in the exclusion of 1074 studies, identifying 155 potentially relevant ones. After retrieving the 130 studies and evaluating their eligibility, 82 were excluded by the exclusion criteria. Finally, 51 studies were selected for the systematic review (Fig. 1).

### Terminology for HFF Types

The studies analysed used the following synonymic denominations.

1. ICF: subcapital, cervical, femoral neck and medial fractures.
2. ECF: intertrochanteric, pertrochanteric, trochanteric, subtrochanteric, and lateral fractures.

To provide a clearer understanding, Figure 2 summarises the locations of HFF types, and the List of Terms used per study can be found in Supplementary Table A1.

### Focus of the Selected Studies

78.4% of the studies (40/51) had as their main objective to study differences between ICF and ECF (Supplementary Table A1).

### Bibliometrics

The bibliometric analysis revealed: 26,067 general publications on hip fragility fractures in older people. 50% of these articles were published in the last 8 years (approximately 1,630 publications per year, excluding 2024). 581 systematic reviews on HFF in older people. 50% of these reviews were published in the last 4 years (72 studies per year, excluding 2024). 1 systematic review [21] on the differences between ICF and ECF, with 51 articles published in the last 40 years (just over one per year).

**Figure 3.**
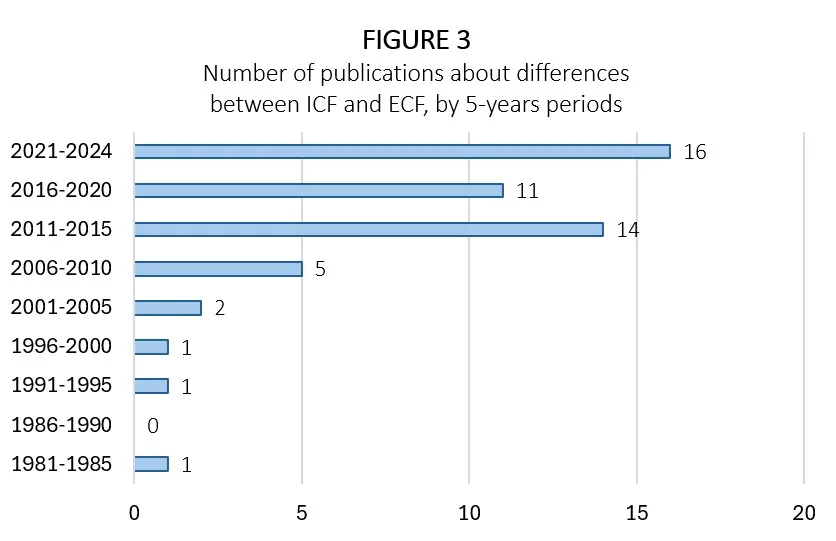
Publication timeleine of selected works (1981-2024).

Regarding publication dates, the period studied spanned 1981 to 2024 (44 years). Initially, between 1981 and 1999, there were few publications. Between 2001and 2010, an increase in the number of studies was observed. Beginning in 2011, growth intensified, peaking between 2021 and 2024 with 16 publications. More than half of the studies was published in the last nine years. In other words, during the last 25% of the studied period, 50% of the papers was published (Fig. 3).

### Geographic distribution of HFF Types

The selected works were conducted across 19 countries over 5 continents: Europe (8 countries), Asia (6 countries), North America (2 countries), South America (2 countries), and Oceania (1 country) (Supplementary Table A1). In a total of 129,075 subjects, ICF presented a frequency of 53%, and ECF 47%. Geographical variations were observed regarding frequencies. ICF highest values were found in Finland (65%), whilst ECF highest values were found in Chile (67%).

**Figure 4.**
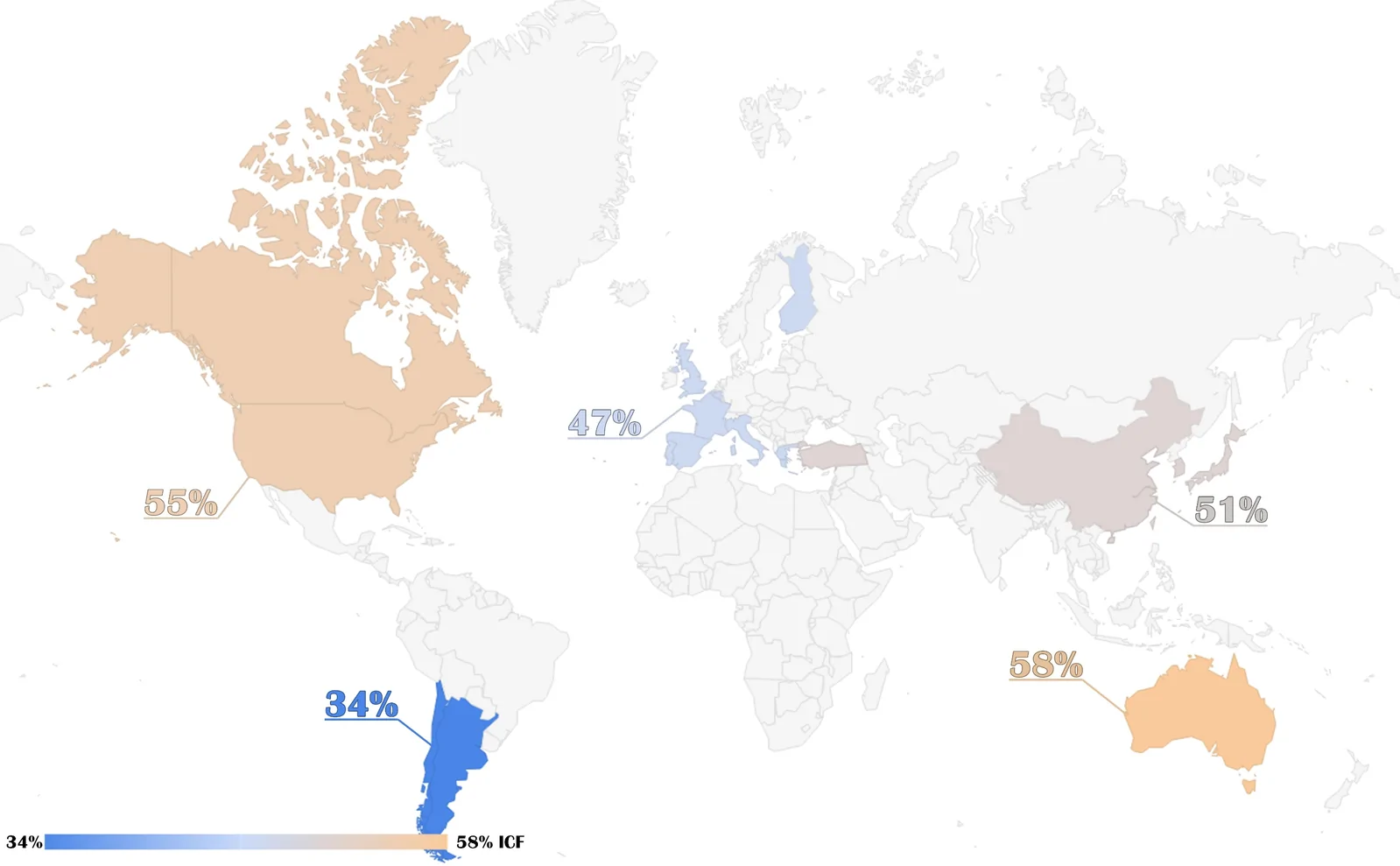
**Global frequencies (%) of ICF across continents**. The more orange the colour, the more frequent the intracapsular hip fractures are. The more blue the colour, the less frequent the intracapsular hip fractures are.

When grouped by continents, European, Asian, and North American countries showed an almost equal proportion of fracture types, with a slight tendency to either ICF or ECF (47%, 51%, and 55% ICF, respectively). On the other hand, all South American countries always presented a higher frequency of ECF (34% ICF) and Australia by itself showed a moderate majority of ICF (58% ICF) (Fig. 4).

### Differences in Parameters

The parameters were extracted from the selected studies to find which were the most evaluated and how often their values were found to be significantly different between the two types of HFF. The number of parameters addressed by work varies between 1 and 14. Most works (72.5%) only measured 1 (*n = 21*) or 2 (*n = 16*) parameters. The average number of parameters measured per work was 2,3. Table 1 summarises the number of subjects, studies, and measurements involved in each parameter. In the Supplementary Table A2 shows the full list of which and how many parameters were measured in each work.

**Table 1:** Studies with significative differences between ICF and ECF (N, %).

Variable Group	Variable	N subjects (%)	N studies (%)	N significant studies (%)

**Demographic**	Age	127322 (98,6)	44 (86,3)	29 (65,9)
	Sex	8730 (6,8)	22 (43,1)	2 (9,1)

**Etiological**	Creatinine	953 (0,7)	3 (5,9)	0 (0)
	Sodium	1562 (1,2)	2 (3,9)	0 (0)
	Potassium	1562 (1,2)	2 (3,9)	0 (0)
	Haemoglobin	2373 (1,8)	4 (7,8)	4 (100)
	Vitamin D	3450 (2,7)	12 (23,5)	4 (33,3)
	Vitamin B12	1341 (1)	2 (3,9)	1 (50)
	Albumin	2647 82,1)	4 (7,8)	2 (50)
	Calcium (c)	1565 (1,2)	4 (7,8)	0 (0)
	PTH	1963 (1,5)	7 (13,7)	4 (47,1)
	TSH	761 (0,6)	1 (2)	0 (0)
	Iron	761 (0,6)	1 (2)	0 (0)
	Transferrin	1085 (0,8)	2 (3,9)	0 (0)
	SAT	761 (0,6)	1 (2)	0 (0)
	eGFR	761 (0,6)	1 (2)	0 (0)

**Therapeutic**	Surgery type	2801 (2,2)	6 (11,8)	6 (100)

**TOTAL**		129075	51	

(c): Calcium corrected according to albumin levels; PTH: Parathormone; TSH: Thyroid Stimulating Hormone; SAT: Percentage of saturation of Transferrin; eGFR: estimated Glomerular Filtration Rate (calculated according to MDRD-6 formula)

**Table 2:** Caption: Comparison between ICF and ECF: Meta-analysis, majority trend and overall agreement.

Variable	ICF	ECF	N studies	>	NS	<	Majority trend	Agreement (%)

**Age (years)**	80,5	83	29	16	10	3	>	62
**Female age (years)**	81,8	83,8	17	12	5	0	>	71
**Male age (years)**	79,9	78,9	7	0	6	1	NS	86
**Sex (Female %)**	77,4	75,5	22	0	20	2	NS	91

**Creatinine (mg/dl)**	1,03	1,07	3	0	3	0	NS	100
**Sodium (mM/l)**	137	136	2	0	2	0	NS	100
**Potassium (mM/l)**	3,99	3,9	2	0	2	0	NS	100
**Haemoglobin (g/dl)**	12,8	11,9	4	0	0	4	<	100
**Vitamin D3 (ng/ml)**	17,3	16,9	12	1	8	3	NS	67
**Vitamin B12 (pg/ml)**	480	438	2	0	1	1	<	50
**Albumin (g/dl)**	3,68	3,51	4	0	2	2	<	50
**Calcium (c) (mg/dl)**	9,18	9,19	4	0	4	0	NS	100
**PTH (pg/ml)**	62,6	76	7	4	3	0	>	57
**TSH (µIU/ml)**	1,7	1,4	1	0	1	0	NS	-
**Iron (µIU/ml)**	26,8	31,8	1	0	1	0	NS	-
**Transferrin (mg/dl)**	186	186	2	0	2	0	NS	100
**SAT (%)**	10,7	12,5	1	0	1	0	NS	-
**eGFR (ml/min)**	65,8	64,3	1	0	1	0	NS	-

**Overall Agreement (%)**								77

>: Values significantly higher in ECF (p<0,05); <: Values significantly lower in ECF (p<0,05); NS: Not significant; (c): Calcium corrected according to albumin levels; PTH: Parathormone; TSH: Thyroid Stimulating Hormone; SAT: Percentage of saturation of Transferrin; eGFR: estimated Glomerular Filtration Rate (calculated according to MDRD-6 formula)

**Figure 5.**
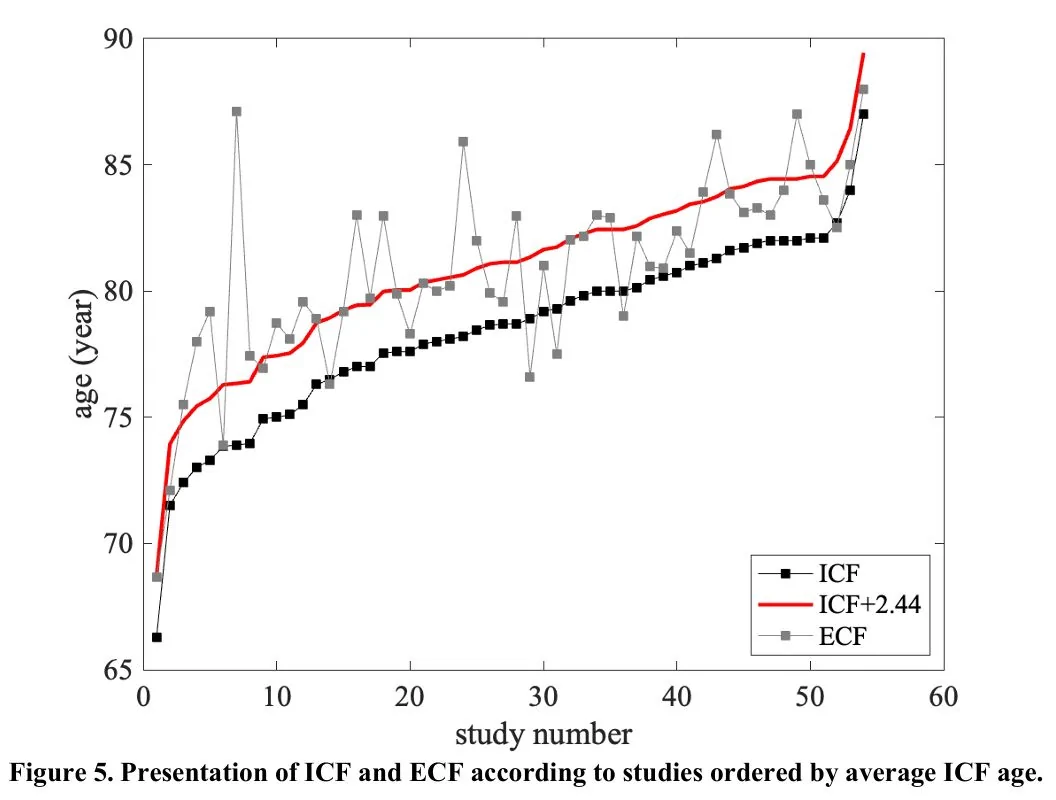
Presentation of ICF and ECF according to studies ordered by average ICF age.

Age was the most measured parameter, recorded in 98.6% of subjects and included in 86.3% of studies, with 65.9% of these finding it statistically significant (p<0.05) [9, 16-18, 22-45]. In Figure 5 and Supplementary Figure 1, it is possible to observe these significant values, consisting in the fact that subjects with ECF are consistently about two and a half years older than subjects with ICF throughout the entire timeline.

Sex, though measured in only 6.8% of subjects, was analysed in 22 studies, with a lower significance rate of 9.1% [46, 47].

CRiF-BD parameters: 1) Vitamin D3 was the most frequently studied one (23.5% of studies), with 33.3% (4/12) of these finding it statistically significant [27, 48-50]. 2) Haemoglobin was the most consistent, with 100% significance rate in all 4 studies that analysed it [36, 45, 50, 51]. 3) PTH was significant in 57.1% (4/7) of studies [27, 31, 36, 52], albumin in 50% (2/4) [36, 45], vitamin B12 in 50% (1/2) [53]. 3) Calcium, TSH, iron, transferrin, percentage of saturation of transferrin, and glomerular filtration rate, showed no statistical significance in the limited number of studies that measured them (Supplementary Fig. 2).

Surgery type was examined in 6 studies (11.8%), all of which found it statistically significant [6, 8, 9, 39, 42, 54].

### Meta-analysis

Results reported in each work were gathered, reporting the mean values of the parameters for both HFF types. In the Supplementary Table A3 shows the list of results gathered per measurement that were found significant. For each parameter, the results were averaged using Equation 1. The trends are considered for each study and parameter, and they are summarised into an overall agreement obtained with Equations 2 and 3. The weighted mean of each parameter, the count of trends, their majority trend, and the overall agreement are showed in Table 2.

Age was evaluated separately for mixed, female, and male cohorts: 1) in mixed cohorts, 16 out of 26 studies (62%) showed that average age of subjects was significantly higher in ECF (ICF=80.5 vs ECF=83.0). 2) The same trend occurs in 12 out of the 17 studies (71%) in female cohorts (ICF=81.8 vs ECF=83.8). 3) Meanwhile, most of male exclusive cohorts showed no significant differences (86%), with only 1 study showing a significant but opposite trend.

CRiF-BD (Table 1, Table 2, Table A3): 1) Serum Haemoglobin levels were found significantly lower in subjects with ECF (ICF=12.8 vs ECF=11.9, g/dL) in all 4 studies (100%). 2) Albumin levels were also found significantly lower in ECF (ICF=3.68 vs ECF=3.51, g/dL) in 2 out of 4 studies (50%). 3) PTH was found significantly higher in ECF (ICF=62.6 vs ECF=76.0, pg/mL) in 4 out of 5 studies (57%). 4) Other parameters such as corrected vitamin D3, calcium, sodium, potassium, creatinine, iron, TSH, transferrin, and saturation percentage of transferrin were found mostly not significant.

The overall agreement was found to be 77%, indicating a moderate level of consistency across the studies regarding the significance of these parameters between ECF and ICF groups. It should be remarked that there are few studies for each type of variable, and they tend to be heterogeneous. This situation, with the exception of age, limited the analytical procedures that could be used, due to the need to adequately control the risk of bias.

## DISCUSSION

General flow of analysis: High heterogeneity was observed among the studies, with a notable scarcity of studies with more than two variables (Fig. 1, Fig. 3, Table 1). Each study used very different methods, time points, and records to obtain their data, and most of them did not publish the row data. This situation considerably limited the possibilities of addressing some analyses (e.g., multivariate analysis, meta-regression, sensitivity analysis), even as exploratory solutions, due to the risk of bias or spurious explanations. On the other hand, the physiological interdependence between variables is well studied and well understood. Therefore, physiologic relations emerge as an interesting possibility to explain the statistical relationships found between variables and can be considered a starting point for formulating hypotheses for future lines of research.

From a bibliometric perspective, there are few scientific publications related to the topic of whether or not ICF and ECF are distinct nosologic entities (Fig. 3). This is especially true when compared to the frequency of publications in other related research areas, such as hip fractures in general. This could mean that this topic has not yet aroused greater interest among researchers. In turn, this may be because the implications that these differences could have, especially in the area of fragility fracture prevention, have not been adequately perceived by researchers. Regardless, this is an underexplored topic, one that clearly requires further research and targeted reporting.

In another matter, it is interesting to note the widespread synonymy found about HFF (Fig. 2). It is advisable a terminological agreement in research, referring to "intracapsular" or "extracapsular" hip fragility fractures. This will facilitate term searches and provide greater conceptual clarity for researchers and clinical professionals, both physicians and surgeons. It will also be especially important in quality-related fields, such as orthogeriatric co-management, health policy formulation, and healthcare resource management.

Regarding the overall frequency of both types of fractures worldwide (Fig. 4), our results show a difference in favour of ICF. This, however, should be considered with caution, as the studies included in this review did not primarily look for determine the incidence of hip fractures. Indeed, the registry systems used are usually local and pursue other objectives. Even when studies sought differences between ICF and ECF, the incidence of each becomes irrelevant for comparing similar frequencies. Furthermore, this systematic review did not consider many incidence studies because they were out-of-scope of our objectives. As a complementary consideration, studies on the differential incidence of HFF are lacking in many countries.

Despite these reasons, it is interesting to consider that if both hip fractures were two manifestations of the same disease entity, the frequency differences between them would not be expected to be significant. This could be stated as a point of attention to recommend that future incidence studies consider separating the two types of hip fragility fractures to obtain data on this matter.

Regarding the geographic location of hip fractures (Fig. 4, Table A1), the results should be interpreted considering that, in this review, geographic location was an indirectly measured variable, assuming that ‘*all subjects in each study come from the same country*.’ Furthermore, most of the samples used in the included studies did not primarily seek to provide epidemiological responses, either at the regional or national level. Because of this last, most studies did not presented adjustments for population's age structure. Therefore, the frequencies presented should not be considered representative of incidence trends. Given the high importance of hip fractures at the organisational and economic levels of health, it is very important to calculate incidence rates for future publications on hip fractures, even if their primary objectives do not include epidemiological aspects.

Even taking these considerations into account, it is interesting to note that significant differences emerge when comparing the frequencies of studies from Europe + South America, which primarily show ECF, and Oceania + North America, which primarily show ICF. Asia showed no differences between the two HFF.

It is quite possible that a ‘continental’ approach of HFF geographical distribution is not entirely accurate for understanding the differences between ICF and ECF. In fact, continental averages can be misleading, as there are important differences between populations within each continent. For example, China (primarily ECF) and Japan (primarily ICF) in Asia, and Greece (primarily ECF) and Finland (primarily ICF) in Europe. These differences within one same continent could be related to nutritional, environmental, and genetic variables between populations, and research about it should be addressed including other variables. Different maps could be generated including these issues.

While it might be risky to consider these results as totally valid from an etiological perspective, it cannot be ignored that there are countries and continents in which the differences shown by the studies were very marked. Again, if HFFs were a single nosological condition, these differences in frequency would be expected to be non-significant. Therefore, the separation of both types of HFFs should be considered in future studies of frequency and incidence, not only globally but also by country. For now, it is a variable available in the hip fracture registry systems of countries that have them.

Regarding demographic parameters, age (Table 1, Table 2, Fig. 5, Table A2) frequently presented significant differences: 65.9% of the studies analysed, both in mixed and exclusively female cohorts, concluded that subjects with ECF were significantly older than those with ICF [9, 16-18, 22-45]. Differences in age at onset suggest that ICF and ECF may differ in both, the rate at which bone damage develops, and the intensity of the bone damage required to produce a fracture: for ICF to develop, bone damage would be more rapid and/or more severe, which would explain its tendency to appear at younger ages. ECF, on the other hand, would require more time, as the damage would occur more slowly and/or less intensely, and therefore would tend to appear at older ages.

Even though this is an attractive explanation, this relationship is not clear in exclusively male cohorts [16, 17, 29, 34, 55, 56]. In one study even there were an inverse relationship [40].

Age-related differences found between sexes may be also due to bone-metabolic differences: in women, peak bone mass is reached around age 18 and is lower than in men. Oestrogens play a fundamental role in the genesis, maintenance, and replacement of bone mass. Oestrogens decrease dramatically during menopause, a programmed and objective event that occurs between 45 and 55 years of age in women. On the other side, in men, maximum bone mass is reached around 21 years of age, is higher than in women, and Androgens are responsible (more than Oestrogens) for its genesis, maintenance, and replacement. The decrease in androgens occurs heterogeneously in the male population, in a phenomenon called andropause, which occurs at highly variable ages and is directly related to fatty infiltration of muscles and bones, rather than to bone mass loss [45]. Therefore, age differences between men and women with respect to the type of HFF will be much more marked and easier to objectify in the female population, which could partly explain the results found.

The second most frequently evaluated parameter was sex (Table 1, Table 2, Table A2). Although most studies found no significant relationship with HFF type [6, 8, 9, 16, 18, 22, 23, 26, 28, 35, 36, 38, 39, 43, 45, 51, 52, 57, 58, 59], two studies significantly associated female sex with ICF [46, 47]. This could be supported by a metabolic cause related to the speed of calcium loss during menopause and climacteric: 30% of menopausal women experience rapid bone loss, while the remaining 70% experience slow bone loss. However, many elements are involved in this dynamic, which act as strong regulators of the rate of bone loss during this process: previous levels of bone mineralisation and muscle mass, number of pregnancies, coexistence with smoking, alcoholism, or use of osteo-damaging drugs such as corticosteroids and antiepileptics, and much more [60].

Thus, the influence of the duration of exposure to risk/protective factors, and the level of resistance of the female and male organism to damage, emerge as a field of interest in the study of the generation of one or another type of HFF.

CRiF-BD are tests with clinical objectives that can be routinely performed in hospitals where patients with HFF are admitted. They have interest in perioperative Orthogeriatrics due to their relationship with bone damage.

Fourteen variables of this type were found in the analysed studies (Table 1, Table 2, Table A3). However, no studies included some important CRiF-BDs such as magnesium [61], thyroid hormones [62], and prothrombin [63]. Furthermore, few studies considered more than four CRiF-BDs [36, 51, 52, 45], and the most analysed CRiF-BDs were the same in most studies (vitamin D3 and parathyroid hormone).

No multivariate analysis of physiologically related variables was found. This is important because, in the body, each variable is always related to others. For example, vitamin D3 enables the absorption of calcium from the intestine into the blood, and blood calcium levels tightly regulate the activity of parathyroid hormone. Therefore, analysing these three variables together would be of great interest.

It is important to note that each study used very different methods, moments and registries to obtain their data. As physiological relationships are well studied and understood, they can therefore be considered a starting point for formulating hypotheses for future lines of research in this field. Physiological interdependence between variables emerges, then, as the first possibility for explaining the relationships and statistical significance founded in the meta-analysis. Exploratory solutions such as meta-regression or multivariate analysis failed due to the lack of data, the great heterogeneity among existing data, and the scarcity of studies on more than two variables. This situation could therefore generate significant bias or even false explanations. Therefore, we consider ethical maintaining only physiological explanations as the first option of explanation of our results.

Next, we will discuss CRiF-BD one by one:

Haemoglobin: haemoglobin relates to other variables in the following way: iron is essential for haemoglobin synthesis. Transferrin iron saturation is a good measure of the amount of iron in the body. When this saturation falls below 20%, erythropoietin, a kidney hormone that stimulates the bone marrow to produce haemoglobin, loses its effectiveness. It is transported by a protein called transferrin, which delivers it to erythroblasts. Erythroblasts are precursor cells to red blood cells and use this iron to produce haemoglobin. Haemoglobin is essential for gas exchange (oxygen and carbon dioxide) at the cellular level and is found within red blood cells, which are the cells responsible for this gas exchange. Haematocrit is the measure of the percentage of red blood cells. Thus, when there is low iron, transferrin saturation is lower, fewer red blood cells are produced, there is less haemoglobin in them, and the haematocrit is lower. This is known as iron deficiency anaemia. And iron deficiency anaemia is an independent risk factor for bone damage [14].

Now, it must be considered that intracapsular and extracapsular zones have differences in blood supply: the intracapsular zone has significantly lower blood supply than the extracapsular zone [7]. Thus, the intracapsular zone is constantly subjected to lower oxygen tensions and greater difficulties in eliminating CO2. In other words, cells in the intracapsular zone have a harder time obtaining adequate oxygen concentrations for survival, making it a more fragile and sensitive area to decreased oxygen in general circulation. Because of this, it is possible that the resistance of the intracapsular zone to anaemia is lower, and that is why ICF occurs with higher haemoglobin concentrations, higher haematocrits, higher transferrin iron saturations.

And at younger ages, as well: It is important to note that for anaemia to have an impact on bone, it needs time, so this explanation applies to chronic anaemias. In other words, this mechanism explains bone damage over time and is consistent with what is seen in relation to the ‘age’ parameter.

However, it does not necessarily imply that anaemia causes different types of bone damage according to each type of fracture: it opens up the possibility that both fractures originate from the same type of bone damage but with different responses depending just on the area of the bone. An interesting way to verify this could be bone biopsy. The study of cases of subjects with a contralateral fracture, occurring time after the first, could also provide interesting explanations.

Albumin levels were also significant in 2 out of 4 studies (ICF=3.68 vs ECF=3.51, g/dL) [36, 45]. Albumin is responsible for the transport of 60% of the plasma calcium to the non-calcified bone matrix, through circulation. In this way, plasma albumin and calcium levels are closely linked to the point that plasma calcium values must be corrected according to albumin levels. Although the corrected plasma calcium levels have not been significant in the studies analysed, it can be considered at least in part that, since albuminemia is significant, calcium is somehow represented in this significance. Indeed, the studies consider these two variables in isolation. On the other hand, low levels of plasmatic albumin (≤3,5g/dL) are a prevalent condition in older people with hip fractures, and it is associated with slower recovery of function and quality of life after surgery [64].

For its part, vitamin B12 is essential to eliminate homocysteine, a terminal product of metabolism, which is neuro- and osteo-injurious [65-67]. However, there are just 2 studies using this CRiF-BD, one without differences (36), and the other (53) showing that subjects with ECF had lower vitamin B12 values than those with ICF.

The explanation for these differences may be similar to that proposed to explain those related to haematocrit and transferrin saturation: the smaller vascular network present in the intracapsular zone. In other words, for the intracapsular zone to become fragile enough to cause a fracture, serum levels of any CRiF-BD must decrease less than for an extracapsular fracture to occur. Better circulation in the extracapsular area would thus become a protective factor, by facilitating the availability of essential elements for bone life, such as oxygen, albumin (calcium), and vitamin B12.

Although this is theoretical, allows for a harmonious, physiologically based explanation for the differences found between CRiF-BD, age and sex. On the other hand, once again, the time of exposure to risk appears to be a central element.

PTH was significant in 4 of 5 studies in mixed cohorts (ICF = 62.8 pg/mL vs ECF = 77.1 pg/mL) [27, 31, 36, 52]. Vitamin D3 only varied in the female cohort, being significant in 2 of 4 studies and lower in ECF (ICF = 17.3 ng/mL vs ECF = 16.9 ng/mL) [48, 50]. PTH and vitamin D3 levels have calcium as a common denominator: if vitamin D3 levels are insufficient, there will not be enough calcium in the plasma, which can be life-threatening. To prevent this from happening, parathyroid hormone is activated, which allows the release of calcium from the bone skeleton. Thus, at lower vitamin D3 levels, higher PTH levels are expected [12, 68, 69]. Therefore, it is consistent that this relationship is also present in the differences found: ICF patients have higher vitamin D3 levels and PTH lower levels, while ECF patients have lower vitamin D3 levels and PTH higher levels.

However, it should be noted that plasmatic vitamin D3 levels do not yet generate definitive agreement regarding its role in the differences between ICF and ECF, and there are studies with contradictory values. Studies with a larger N and multivariate analysis are needed to define the role of this variable [8, 36].

Regarding kind of surgical treatment differences (Table 1, Table 2, Table A2), there is strong consistency and statistical significance between ICF and treatment with arthroplasty, and between ECF and treatment with osteosynthesis. On the other hand, opposing treatments are rare in both groups: ECF is rarely treated with arthroplasty, which is usually related to degenerative changes in the synovial joint; and when ICF is treated with osteosynthesis systems, the prognosis is generally significantly negative. This difference is also statistically significant. Therefore, among therapeutic aspects, the type of surgical procedure, with all six studies reporting significant differences [6, 8, 9, 39, 42, 54], is one of the most relevant variables when discussing the differences between ICF and ECF.

Based on these results, we propose that the variables studied be classified according to the presence or absence of differences between ICF and ECF as follows:

‘A’ Variables: Age, haemoglobin, PTH, and type of surgical treatment.

The presence of differences between ICF and ECF with respect to these variables is clear and conclusive.

‘B’ Variables: Sex and serum calcium.

The absence of differences between ICF and ECF with respect to these variables is clear and conclusive.

‘C’ Variables: This is the largest group. It includes variables for which the existence or absence of differences between ICF and ECF has not yet been conclusively elucidated.

The lack of conclusiveness may be due to the absence of specific studies on the subject (e.g., haematocrit, thyroid hormones), or to the scarcity of studies (geographic location, TSH, vitamin B12, iron, transferrin, transferrin saturation, creatinine, glomerular filtration rate, sodium, potassium), to conflicting results (vitamin D3), or to the scarcity of positive results (albumin).

‘C’ variables should be studied in greater depth, with specific designs that facilitate obtaining better samples, with larger *N*s, with ideally multicentric studies, and performing better analyses, ideally multivariate.

Definitive differences between intracapsular (ICF) and extracapsular (ECF) hip fragility fractures would provide evidence for different preventive approaches, which is of great importance for patients, organizations, and resource allocation. Despite the importance of distinguishing between these fracture types, few studies exist. Therefore, we encourage researchers to further explore this topic through multivariate analyses of various CRiF-BDs, genetic variables, and bone biopsies to better understand these differences and optimise preventive strategies.

### Limitations of the present study:

This study has several limitations. It may not include all the available literature on the topic. The lack of standardised methodologies in obtaining data, the lack of geographic representation, and the lack of data about standardised HFF incidence rate for each studied region, further limit both, the analytical procedures and the generalisability of the findings. Geographic and demographic variability was assessed indirectly, which reduced representativeness. Some studies were not designed to assess fracture frequency, and key variables such as haemoglobin and albumin were analysed in isolation without multivariate approaches. Finally, even though physiologic explanations are strong and interesting, there are potential residual confounding that should take into account at the moment of more definitive interpretations, as comorbidities, medication use, and socioeconomic status. Future research in this area would include these variables as well.

### Strengths of the present study:

Its statistical power is high due to the large sample size. In fact, only one previous review [21] considered 19 publications and 4 CRiF-BD, fewer than the present review, which includes 51 publications and 14 parameters. The results allow us to clearly identify the need for studies with more than 10 CRiF-BD and suggest a multivariate analysis, which appears in few of the analysed studies. The statistical methods employed allow us to conclude there are variables that clearly mark differences between ICF and ECF, so it would not be necessary to continue investigating them independently, but they could serve as references. Finally, by emphasising CRiF-BD, the present review encourages the creation of hospital measurement and recording systems for these parameters, which can be used to improve the quality of life of patients with HFF.

### Conclusions

1. Significant differences between ICF and ECF have been observed in epidemiological, demographical, etiological, and therapeutic aspects. There are also some parameters without differences:
  - Epidemiologically, HFF have significant differences in geographic distributions between countries and continents. Europe and South America presented mainly ECF; Oceania and North America mainly ICF; and Asia showed no differences. Even though, there are significant differences between countries of one same continent.
  - Demographically, ICF occurs at significantly lower ages than ECF (81 vs 84 years old). On the other hand, sex did not showed differences between both HFF.
  - Etiologically, there are significant differences in some clinical risk factors for bone disease (CRiF-BD): Haemoglobin and albumin presented higher values in ICF than in ECF. On the other side, plasmatic calcium concentration did not showed differences between both HFF.
  - In the therapeutic field, the type of surgical procedure is one of the strongest variables distinguishing ICF from ECF, with arthroplasty being almost always for ICF and osteosynthesis for ECF.
2. For future research, we recommend:
  - It is especially important achieving agreements in the definition of variables, raw data collection and standardisation, and data analysis:
    1. Separating HFF into ICF and ECF, especially in studies of frequency and incidence.
    2. It is advisable a terminological agreement in research, referring to ‘intracapsular’ or ‘extracapsular’ hip fragility fractures, thus avoiding the widespread synonymy found, which can lead to confusion.
    3. We strongly recommend that HFF researchers include incidence rates for their samples relative to their studied populations, even if the study has different objectives.
    4. Along with this, it is necessary to generate studies using multivariate analysis over more than one CRiF-BD.
  - Histopathologic studies could be needed to elucidate the relation of arterial irrigation with the location of HFF.
  - These results will allow researchers to address the role of pre-fracture markers called CRiF-BD on functional status, quality of life, and mortality, among other variables, by searching for differences between ICF and ECF.

## Supplementary Materials

The Supplementary data can be found online at: www.aginganddisease.org/EN/10.14336/AD.2025.0414.



## Data Availability

The data analysed during the current study can be obtained by contacting the authors.
