# Supplementary material for: Hip Fragility Fractures: One Or Two Pathologies? A Systematic Review and Meta-Analysis of Demographic, Diagnostic and Therapeutic Aspects
Source: Aging Dis. 2026 Jul 25;17(5):2561–76. doi: 10.14336/AD.2025.0414 (PMC13437110; doi:10.14336/AD.2025.0414)
Supplement: Supplementary file 1 [file AD-17-5-2561-s.pdf]

SUPPLEMENTARY DATA

Hip Fragility Fractures: One Or Two Pathologies? A Systematic Review and Meta-Analysis of Demographic, Diagnostic and Therapeutic Aspects

José Luis Dinamarca-Montecinos, Miguel Yáñez, Ana Aguilera, Jean-Gabriel Minonzio

© 2025. Dinamarca-Montecinos JL et al. Published online at http://www.aginganddisease.org/EN/10.14336/AD.2025.0414

SUPPLEMENTARY DATA

Supplementary Table A1. Selected studies in the review grouped by their geographic location and the number of included subjects for both HFF types.

Year	Work	Country	Subjects	ICF	ECF	Comparison	Aim
2017	[58]	China	117	69	48	FN/TR	★
2018	[18]	China	198	101	97	FN/IT	★
2019	[17]	China	625	381	244	FN/TR	★
2019	[16]	China	196	109	87	CV/TR	★
2020	[30]	China	441	266	175	FN/IT	★
2021	[70]	China	40	20	20	FN/TR	★
2023	[26]	China	593	404	189	FN/IT	★
2023	[24]	China	554	314	240	FN/TR	★
		China	2764	1664 (60%)	1100 (40%)		
2011	[59]	Japan	40	22	18	FN/TR	★
2016	[32]	Japan	6131	2826	3305	FN/TR	
2017	[33]	Japan	100	63	37	FN/TR	★
2022	[27]	Japan	310	139	171	FN/TR	
2023	[25]	Japan	252	138	114	FN/TR	
		Japan	6833	3188 (47%)	3645 (53%)		
2012	[47]	Singapore	275	169	106	FN/TR	★
2023	[57]	S. Korea	168	76	92	FN/IT	
2020	[51]	Taiwan	139	86	53	FN/BN,IT,ST	★
2010	[39]	Turkey	248	136	112	FN/IT	★
2023	[23]	Turkey	233	94	139	FN/TR	★
		Turkey	481	230 (48%)	251 (52%)		
**Asia**			**10660**	**5413 (51%)**	**5247 (49%)**		
2007	[71]	Belgium	170	84	86	FN/IT	★
2002	[72]	Finland	36	24	12	CV/TR	
2010	[73]	Finland	57	39	18	CV/TR	★
2012	[37]	Finland	80	49	31	CV/TR	★
		Finland	173	112 (65%)	61 (35%)		
2005	[44]	France	192	108	84	CV/TR	★
2006	[43]	Greece	518	172	346	FN/IT	★
2011	[49]	Greece	53	24	29	CV/TR	★
2019	[31]	Greece	116	56	60	SC/TR	★
2020	[29]	Greece	2258	926	1332	ICF/IT	★
2024	[22]	Greece	70	40	30	FN/TR	★
		Greece	3015	1218 (40%)	1797 (60%)		
2007	[42]	Italy	684	335	349	CV/TR	★
2007	[41]	Italy	429	273	156	FN/TR	★
2013	[74]	Italy	689	307	382	CV/TR	★
2021	[28]	Italy	531	295	236	FN/IT	★
2022	[75]	Italy	363	147	216	Medial/Lateral	

© 2025. Dinamarca-Montecinos JL et al. Published online at http://www.aginganddisease.org/EN/10.14336/AD.2025.0414

SUPPLEMENTARY DATA

		Italy	2696	1357 (50%)	1339 (50%)		
2023	[9]	Portugal	299	103	196	FN/TR,ST	
2012	[38]	Spain	471	265	206	CV/TR	★
2012	[52]	Spain	324	128	196	SC,BC/ST,PT	★
		Spain	795	393 (49%)	402 (51%)		
1992	[45]	UK	1423	726	697	ICF/ECF	★
1999	[56]	UK	310	192	118	CV/TR	★
		UK	1733	918 (53%)	815 (47%)		
**Europe**			**9073**	**4225 (47%)**	**4848 (53%)**		
2010	[40]	Canada	2150	1167	983	SC/IT	
2016	[34]	Canada	102842	56306	46536	SC/IT	★
		Canada	104992	57473 (55%)	47519 (45%)		
1983	[50]	USA	50	24	26	CV/TR	★
2000	[8]	USA	923	427	496	FN/IT	★
2013	[35]	USA	231	113	118	FN/IT	★
2014	[46]	USA	325	167	158	FN/TR	★
2020	[55]	USA	112	51	61	FN/TR	★
		USA	1641	782 (48%)	859 (52%)		
**North America**			**106633**	**58255 (55%)**	**48378 (45%)**		
2014	[76]	Argentina	499	195	304	Medial/Lateral	
2015	[48]	Chile	222	96	126	ICF/ECF	★
2015	[54]	Chile	647	218	429	ICF/ECF	
2015	[6]	Chile	647	218	429	ICF/ECF	★
2022	[53]	Chile	580	161	419	ICF/ECF	
		Chile	1449	475 (33%)	974 (67%)		
**South America**			**1948**	**670 (34%)**	**1278 (66%)**		
**2012**	**[36]**	**Australia**	**761**	**444**	**317**	**CV/TR**	★
**Total**			**129075**	**69007 (53%)**	**60068 (47%)**		**78%**

★: The study had as main objective the comparison between ICF and ECF, FN: Femoral Neck, CV: Cervical, SC: Subcapital, BN: Basal Neck, TR: Trochanteric, IT: Intertrochanteric, ST: Subtrochanteric, PT: Pertrochanteric, ICF: Intracapsular, ECF: Extracapsular.

© 2025. Dinamarca-Montecinos JL et al. Published online at http://www.aginganddisease.org/EN/10.14336/AD.2025.0414

SUPPLEMENTARY DATA

Supplementary Table A2. Count of parameters measured and their statistical significance in each work.

	Parameters																
Work	Age	Sex	Creatinine	Sodium	Potassium	Hb	Vit. D	Vit. B12	Albumin	Calcium	PTH	TSH	Iron	Transferrin	SAT	eGFR	Surgery	Params
Fisher et al. [36]	X	O	O			X	O	O	X	O	X	O	O	O	O	O		14
Cheng et al. [51]	O	O	O	O	O	X			O									7
Larrosa et al. [52]	O	O					O		O	O	X			O				7
Parker et al. [45]	X	O		O	O	X			X									6
Dretakis and Igoumenou [31]	X						O			O	X							4
Chandran et al. [47]	O	X					O			O								4
Dretakis et al. [49]			O				X			O	O							4
Lourenço et al. [9]	X	O															X	3
Kanno et al. [27]	X						X				X							3
Dinamarca-Montecinos et al. [6]	O	O															X	3
Kesmezacar et al. [39]	X	O															X	3
Partanen et al. [72]							O			O	O							3
Fox et al. [8]	X	O															X	3
Lawton et al. [50]	O					X	X											3
Vlachos et al. [22]	X	O																2
Li et al. [26]	X	O																2
Che et al. [57]	O	O																2
Celik et al. [23]	X	O																2
Caffarelli et al. [75]							O				O							2
Arcolin et al. [28]	X	O																2
Zhao et al. [30]	X						O											2
Li et al. [16]	X	O																2
Hu et al. [18]	X	O																2
Zhuang et al. [58]	O	O																2
Mangram et al. [46]	O	X																2
Strike et al. [35]	X	O																2
Riancho et al. [38]	X	O																2
Maeda et al. [59]	O	O																2
Di Monaco et al. [42]	X																X	2
Karagiannis et al. [43]	X	O																2
Kanazawa et al. [25]	X																	1
Huang et al. [24]	X																	1
Dinamarca-Montecinos and Vásquez-Leiva [53]								X										1
Ling et al. [70]	O																	1
Alpantaki et al. [29]	X																	1

© 2025. Dinamarca-Montecinos JL et al. Published online at http://www.aginganddisease.org/EN/10.14336/AD.2025.0414

SUPPLEMENTARY DATA

Su et al. [17]	X																	1
Horii et al. [33]	X																	1
Horii et al. [32]	X																	1
Crilly et al. [34]	X																	1
Dinamarca Montecinos et al. [48]							X											1
Dinamarca-Montecinos et al. [54]																	X	1
Diehl et al. [76]							O											1
Gonnelli et al. [74]	O																	1
Määttä et al. [37]	X																	1
Tanner et al. [40]	X																	1
Pulkkinen et al. [73]	O																	1
Haentjens et al. [71]	O																	1
Gnudi et al. [41]	X																	1
Schott et al. [44]	X																	1
Stewart et al. [56]	O																	1
Greenspan et al. [55]	O																	1
**Mean parameters measured per work**																		**2,3**

X: Statistically significant measurement between type of HFF, O: Non-statistically significant measurement between type of HFF.

Supplementary Table A3. Recompilation of significant results reported in the selected works.

Parameter	Ref	Samples	Females	ICF	ECF	p-value
**Age**	Parker et al. [45]	1423	82%	78	80	<0.001
	Karagiannis et al. [43]	518	72.20%	77.6	79.9	0.001
	Kesmezacar et al. [39]	248	69.35%	77.9	80.3	0.02
	Riancho et al. [38]	471	81.10%	80	83	0.001
	Fisher et al. [36]	761	74.90%	81.7	83.1	0.034
	Strike et al. [35]	231	71.42%	80	82.9	0.002
	Horii et al. [32]	6131	81.20%	82.1	85	0.0000
	Hu et al. [18]	198	77.78%	76.3	78.9	0.014
	Li et al. [16]	196	64.28%	75	78.72	0.005
	Dretakis and Igoumenou [31]	116	58.60%	73.9	87.1	<0.001
	Arcolin et al. [28]	531	81.17%	82.1	83.6	<0.05
	Kanno et al. [27]	310	80%	82	87	<0.001
	Lourenço et al. [9]	299	81%	81.61	83.84	0.02
	Li et al. [26]	593	72%	78.46	81.99	<0.01
	Celik et al. [23]	233	63.90%	79.62	82.03	0.015
	Vlachos et al. [22]	70	72.90%	78.7	82.97	0.05
	Schott et al. [44]	192	100%	81.9	83.3	<0.05
	Di Monaco et al. [42]	684	100%	78.64	79.91	0.04
	Gnudi et al. [41]	429	100%	73	78	0.001
	Tanner et al. [40]	1595	100%	81.1	83.9	<0.001
	Määttä et al. [37]	80	100%	71.5	72.1	0.031
	Crilly et al. [34]	74455	100%	82	84	<0.0001
	Horii et al. [33]	100	100%	78.2	85.9	<0.005
	Li et al. [26]	126	100%	75.51	79.56	0.009
	Zhao et al. [30]	441	100%	77.54	82.98	<0.05
	Alpantaki et al. [29]	1727	100%	80.14	82.15	<0.0001
	Kanazawa et al. [25]	252	100%	81.3	86.2	<0.001
	Huang et al. [24]	554	100%	77.02	79.7	<0.001
	Tanner et al. [40]	555	0%	79.3	77.5	0.05
**Sex**	Mangram et al. [46]	325	-	74	66	<0.001
	Chandran et al. [47]	275	-	79.64	66.67	0.045

SUPPLEMENTARY DATA

**Vit. D**	Kanno et al. [27]	310	80%	11.7	10.7	<0.05
	Dretakis and Igoumenou [31]	53	100%	22.1	14.39	0.015
	Lawton et al. [50]	71	100%	17.3	10.9	<0.01
	Dinamarca Montecinos et al. [48]	222	80.60%	-	+4.52	<0.001
**PTH**	Kanno et al. [27]	310	80%	46.4	50.9	<0.01
	Dretakis and Igoumenou [31]	116	58.60%	70.8	86.1	0.001
	Larrosa et al. [52]	324	85.18%	76	91	0.03
	Fisher et al. [36]	761	74.90%	55.64	75.44	0.001
**Hb**	Cheng et al. [51]	139	74.10%	12.48	11.44	0.002
	Fisher et al. [36]	761	74.90%	12.82	12.18	0.000
	Parker et al. [45]	1423	82%	12.9	11.9	<0.001
	Lawton et al. [50]	71	100%	12.7	10.5	<0.001
**Albumin**	Fisher et al. [36]	761	74.90%	3.60	3.45	0.002
	Parker et al. [45]	1423	82%	3.72	3.52	<0.001
**Vit. B12**	Dinamarca-Montecinos and Vásquez-Leiva [53]	580	79.31%	380	338	0.013

© 2025. Dinamarca-Montecinos JL et al. Published online at http://www.aginganddisease.org/EN/10.14336/AD.2025.0414

SUPPLEMENTARY DATA

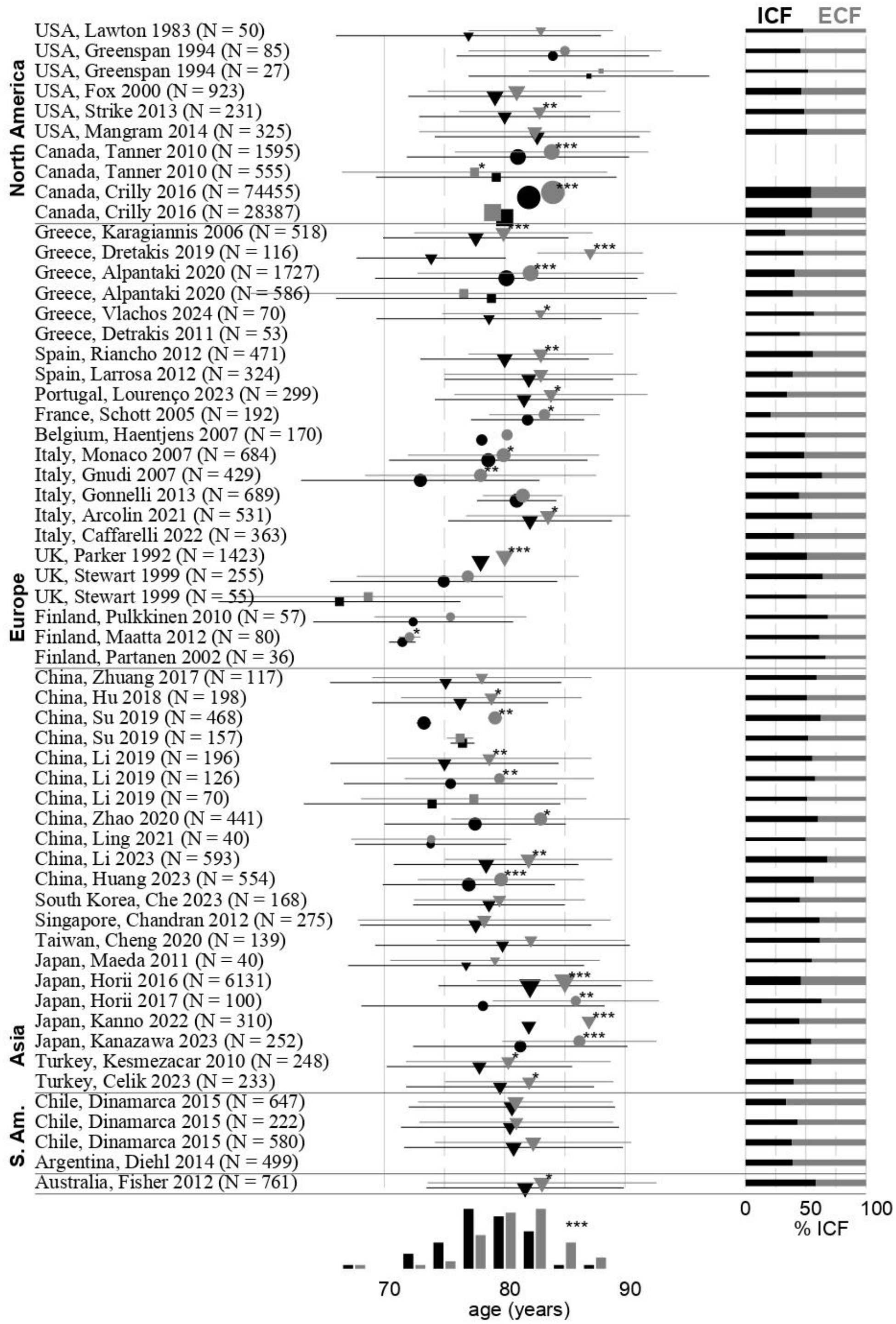

Supplementary Figure 1. age differences between ICF and ECF (Forest plot). The first column indicates the studies, classified by continent, country first author, publication year and patient number. The second column illustrates the age mean, indicated with symbols for women (circle), men (triangle), both (square) and standard deviations (line) for the two groups, ICF (black) and ECF (gray). The number of stars indicate the statistical significance: * ($p < 0.05$), ** ($p < 0.01$) and *** ($p < 0.001$). Finally, the third column indicates the % repartition between the two groups using the same colors: ICF (black) and ECF (gray). The age histogram summary is shown at the bottom of the figure.

© 2025. Dinamarca-Montecinos JL et al. Published online at http://www.aginganddisease.org/EN/10.14336/AD.2025.0414

SUPPLEMENTARY DATA

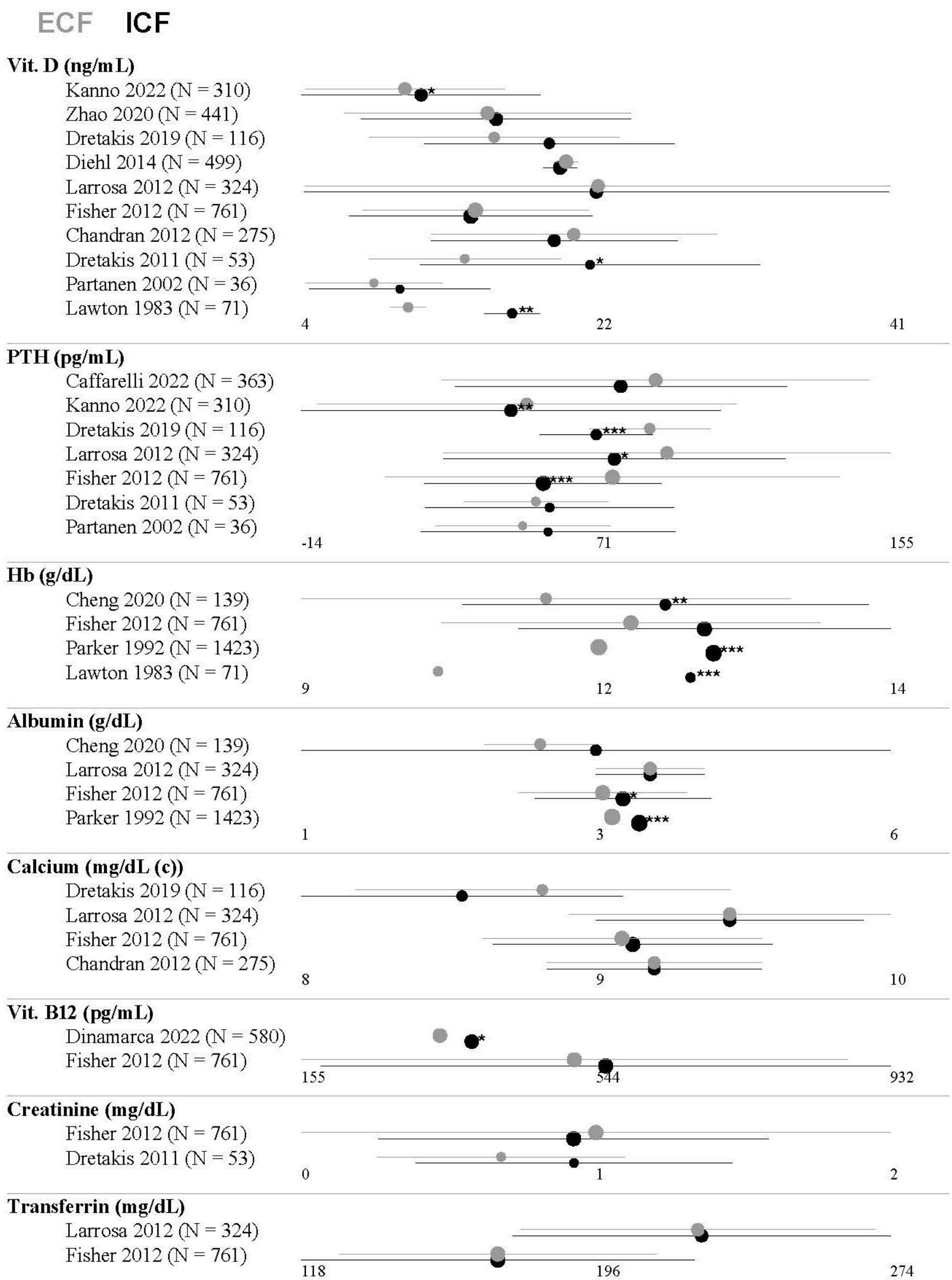

Supplementary Figure 2. blood test differences between ICF and ECF (Forest plot). The first column indicates the blood test paramaters and the studies, classified by publication year, first author, and patient number. The second column illustrates the mean value, indicatesd with a circle and standard deviations (line) for the two groups, ICF (black) and ECF (gray). The number of stars indicate the statistical significance : * ($p < 0.05$), ** ($p < 0.01$) and *** ($p < 0.001$).

© 2025. Dinamarca-Montecinos JL et al. Published online at http://www.aginganddisease.org/EN/10.14336/AD.2025.0414

SUPPLEMENTARY DATA

PRISMA 2020 Checklist

Section and Topic	Item #	Checklist item	Location where item is reported
**TITLE**			
Title	1	Identify the report as a systematic review.	https://doi.org/10.17605/OSF.IO/2U67V
**ABSTRACT**			
Abstract	2	See the PRISMA 2020 for Abstracts checklist.	https://doi.org/10.17605/OSF.IO/2U67V
**INTRODUCTION**			
Rationale	3	Describe the rationale for the review in the context of existing knowledge.	https://doi.org/10.17605/OSF.IO/2U67V
Objectives	4	Provide an explicit statement of the objective(s) or question(s) the review addresses.	https://doi.org/10.17605/OSF.IO/2U67V
**METHODS**			
Eligibility criteria	5	Specify the inclusion and exclusion criteria for the review and how studies were grouped for the syntheses.	https://archive.org/details/osf-registrations-2u67v-v1
Information sources	6	Specify all databases, registers, websites, organisations, reference lists and other sources searched or consulted to identify studies. Specify the date when each source was last searched or consulted.	https://archive.org/details/osf-registrations-2u67v-v1
Search strategy	7	Present the full search strategies for all databases, registers and websites, including any filters and limits used.	https://doi.org/10.17605/OSF.IO/2U67V
Selection process	8	Specify the methods used to decide whether a study met the inclusion criteria of the review, including how many reviewers screened each record and each report retrieved, whether they worked independently, and if applicable, details of automation tools used in the process.	https://archive.org/details/osf-registrations-2u67v-v1
Data collection process	9	Specify the methods used to collect data from reports, including how many reviewers collected data from each report, whether they worked independently, any processes for obtaining or confirming data from study investigators, and if applicable, details of automation tools used in the process.	https://archive.org/details/osf-registrations-2u67v-v1
Data items	10a	List and define all outcomes for which data were sought. Specify whether all results that were compatible with each outcome domain in each study were sought (e.g. for all measures, time points, analyses), and if not, the methods used to decide which results to collect.	https://archive.org/details/osf-registrations-2u67v-v1
	10b	List and define all other variables for which data were sought (e.g. participant and intervention characteristics, funding sources). Describe any assumptions made about any missing or unclear information.	https://archive.org/details/osf-registrations-2u67v-v1
Study risk of bias assessment	11	Specify the methods used to assess risk of bias in the included studies, including details of the tool(s) used, how many reviewers assessed each study and whether they worked independently, and if applicable, details of automation tools used in the process.	https://archive.org/details/osf-registrations-2u67v-v1
Effect measures	12	Specify for each outcome the effect measure(s) (e.g. risk ratio, mean difference) used in the synthesis or presentation of results.	https://archive.org/details/osf-registrations-2u67v-v1
Synthesis methods	13a	Describe the processes used to decide which studies were eligible for each synthesis (e.g. tabulating the study intervention characteristics and comparing against the	https://archive.org/details/osf-registrations-2u67v-v1

		planned groups for each synthesis (item #5)).	
	13b	Describe any methods required to prepare the data for presentation or synthesis, such as handling of missing summary statistics, or data conversions.	https://archive.org/details/osf-registrations-2u67v-v1
	13c	Describe any methods used to tabulate or visually display results of individual studies and syntheses.	https://archive.org/details/osf-registrations-2u67v-v1
	13d	Describe any methods used to synthesize results and provide a rationale for the choice(s). If meta-analysis was performed, describe the model(s), method(s) to identify the presence and extent of statistical heterogeneity, and software package(s) used.	https://archive.org/details/osf-registrations-2u67v-v1
	13e	Describe any methods used to explore possible causes of heterogeneity among study results (e.g. subgroup analysis, meta-regression).	https://archive.org/details/osf-registrations-2u67v-v1
	13f	Describe any sensitivity analyses conducted to assess robustness of the synthesized results.	https://archive.org/details/osf-registrations-2u67v-v1
Reporting bias assessment	14	Describe any methods used to assess risk of bias due to missing results in a synthesis (arising from reporting biases).	https://archive.org/details/osf-registrations-2u67v-v1
Certainty assessment	15	Describe any methods used to assess certainty (or confidence) in the body of evidence for an outcome.	https://archive.org/details/osf-registrations-2u67v-v1
**RESULTS**			
Study selection	16a	Describe the results of the search and selection process, from the number of records identified in the search to the number of studies included in the review, ideally using a flow diagram.	https://archive.org/details/osf-registrations-2u67v-v1
	16b	Cite studies that might appear to meet the inclusion criteria, but which were excluded, and explain why they were excluded.	https://archive.org/details/osf-registrations-2u67v-v1
Study characteristics	17	Cite each included study and present its characteristics.	https://archive.org/details/osf-registrations-2u67v-v1
Risk of bias in studies	18	Present assessments of risk of bias for each included study.	https://archive.org/details/osf-registrations-2u67v-v1
Results of individual studies	19	For all outcomes, present, for each study: (a) summary statistics for each group (where appropriate) and (b) an effect estimate and its precision (e.g. confidence/credible interval), ideally using structured tables or plots.	https://archive.org/details/osf-registrations-2u67v-v1
Results of syntheses	20a	For each synthesis, briefly summarise the characteristics and risk of bias among contributing studies.	https://archive.org/details/osf-registrations-2u67v-v1
	20b	Present results of all statistical syntheses conducted. If meta-analysis was done, present for each the summary estimate and its precision (e.g. confidence/credible interval) and measures of statistical heterogeneity. If comparing groups, describe the direction of the effect.	https://archive.org/details/osf-registrations-2u67v-v1
	20c	Present results of all investigations of possible causes of heterogeneity among study results.	https://archive.org/details/osf-registrations-2u67v-v1
	20d	Present results of all sensitivity analyses conducted to assess the robustness of the synthesized results.	https://archive.org/details/osf-registrations-2u67v-v1
Reporting biases	21	Present assessments of risk of bias due to missing results (arising from reporting biases) for each synthesis assessed.	https://archive.org/details/osf-registrations-2u67v-v1
Certainty of evidence	22	Present assessments of certainty (or confidence) in the body of evidence for each outcome assessed.	https://archive.org/details/osf-registrations-2u67v-v1

© 2025. Dinamarca-Montecinos JL et al. Published online at http://www.aginganddisease.org/EN/10.14336/AD.2025.0414

SUPPLEMENTARY DATA

**DISCUSSION**			
Discussion	23a	Provide a general interpretation of the results in the context of other evidence.	https://archive.org/details/osf-registrations-2u67v-v1
	23b	Discuss any limitations of the evidence included in the review.	https://archive.org/details/osf-registrations-2u67v-v1
	23c	Discuss any limitations of the review processes used.	https://archive.org/details/osf-registrations-2u67v-v1
	23d	Discuss implications of the results for practice, policy, and future research.	https://archive.org/details/osf-registrations-2u67v-v1
**OTHER INFORMATION**			
Registration and protocol	24a	Provide registration information for the review, including register name and registration number, or state that the review was not registered.	https://archive.org/details/osf-registrations-2u67v-v1
	24b	Indicate where the review protocol can be accessed, or state that a protocol was not prepared.	https://archive.org/details/osf-registrations-2u67v-v1
	24c	Describe and explain any amendments to information provided at registration or in the protocol.	https://archive.org/details/osf-registrations-2u67v-v1
Support	25	Describe sources of financial or non-financial support for the review, and the role of the funders or sponsors in the review.	https://archive.org/details/osf-registrations-2u67v-v1
Competing interests	26	Declare any competing interests of review authors.	https://archive.org/details/osf-registrations-2u67v-v1
Availability of data, code and other materials	27	Report which of the following are publicly available and where they can be found: template data collection forms; data extracted from included studies; data used for all analyses; analytic code; any other materials used in the review.	https://archive.org/details/osf-registrations-2u67v-v1

From: Page MJ, McKenzie JE, Bossuyt PM, Boutron I, Hoffmann TC, Mulrow CD, et al. The PRISMA 2020 statement: an updated guideline for reporting systematic reviews. BMJ 2021;372:n71. doi: 10.1136/bmj.n71. This work is licensed under CC BY 4.0. To view a copy of this license, visit https://creativecommons.org/licenses/by/4.0/
